# The rapid-reaction kinetics of an electron-bifurcating flavoprotein, the crotonyl-CoA-dependent NADH:ferredoxin oxidoreductase EtfAB:bcd

**DOI:** 10.1016/j.jbc.2024.107745

**Published:** 2024-09-03

**Authors:** Derek Nguyen, Wayne Vigil, Dimitri Niks, Russ Hille

**Affiliations:** Department of Biochemistry, The University of California, Riverside, California, USA

**Keywords:** electron bifurcation, flavoprotein, rapid-reaction kinetics, electron-transferring flavoprotein, ferredoxin

## Abstract

We have investigated the kinetic behavior of the electron-bifurcating crotonyl-CoA-dependent NADH: ferredoxin oxidoreductase EtfAB:bcd from *Megasphaera elsdenii*. The overall behavior of the complex in both the reductive and the oxidative half-reactions is consistent with that previously determined for the individual EtfAB and bcd components. This includes an uncrossing of the half-potentials of the bifurcating flavin of the EtfAB component in the course of ferredoxin-reducing catalysis, ionization of the bcd flavin semiquinone and the appearance of a charge transfer complex upon binding of the high potential acceptor crotonyl-CoA. The observed rapid-reaction rates of ferredoxin reduction are independent of [NADH], [crotonyl-CoA], and [ferredoxin], with an observed rate of ∼0.2 s^−1^, consistent with the observed steady-state kinetics. In enzyme-monitored turnover experiments, an approach to steady-state where the complex’s flavins become reduced but no ferredoxin is generated is followed by a steady-state phase characterized by extensive ferredoxin reduction but little change in overall levels of flavin reduction. The approach to the steady-state phase can be eliminated by prior reduction of the complex, in which case there is no lag in the onset of ferredoxin reduction; this is consistent with the et FAD needing to be reduced to the level of the (anionic) semiquinone for bifurcation and concomitant ferredoxin reduction to occur. Single-turnover experiments support this conclusion, with the accumulation of the anionic semiquinone of the et FAD apparently required to prime the system for subsequent bifurcation and ferredoxin reduction.

Flavin-based electron bifurcation is a recently recognized form of energy conservation found in many anaerobic archaea and bacteria, allowing them to generate essential low-potential reducing equivalents for a multitude of cellular processes (*i.e.* carbon and nitrogen fixation) although their poised intracellular potential is only modestly reducing (1, 2). Bifurcation involves taking a pair of electrons from a median-potential electron donor and sending them individually into separate low- and high-potential pathways in a manner that is overall thermodynamically favorable, ultimately using them to reduce separate low- and high-potential acceptors, respectively. Median-potential electron donors in known bifurcating systems include NADH and NADPH, high-potential acceptors include crotonyl-CoA, caffeyl-CoA, menaquinone, ubiquinone, or pyruvate, and low-potential acceptors ferredoxin and flavodoxin.

The thermodynamics of flavin-based electron bifurcation are well understood, the process being made possible due to the ability of the isoalloxazine ring of FAD to undergo both one- and two-electron transfer events. FAD can exist in three different quinone states: the fully oxidized quinone state ([Q], FAD), a one-electron reduced semiquinone state ([SQ], the neutral FADH• or anionic FAD•^-^, depending on the pK_a_), and a fully reduced hydroquinone state ([HQ], FADH^-^ at neutral pH). A fundamental characteristic of flavin-based electron bifurcation is that the bifurcating flavin has highly crossed half-potentials, meaning that the half-potential for the SQ/HQ couple is much higher than that for the Q/SQ couple (the midpoint potential for the two-electron process being the average of the two half-potentials). As shown schematically in Figure 1*A*, after reduction to the hydroquinone in a two-electron process and the departure of a first, high-potential electron from the site of bifurcation into a high-potential pathway, a very low-potential FAD•^-^ remains that can either be transferred rapidly into a discrete low-potential pathway or used to reduce a low-potential substrate such as ferredoxin directly. Highly crossed half-potentials have been observed in all known flavin-based bifurcating systems and the condition appears to be an essential property of bifurcating systems (1).

**Figure 1:**
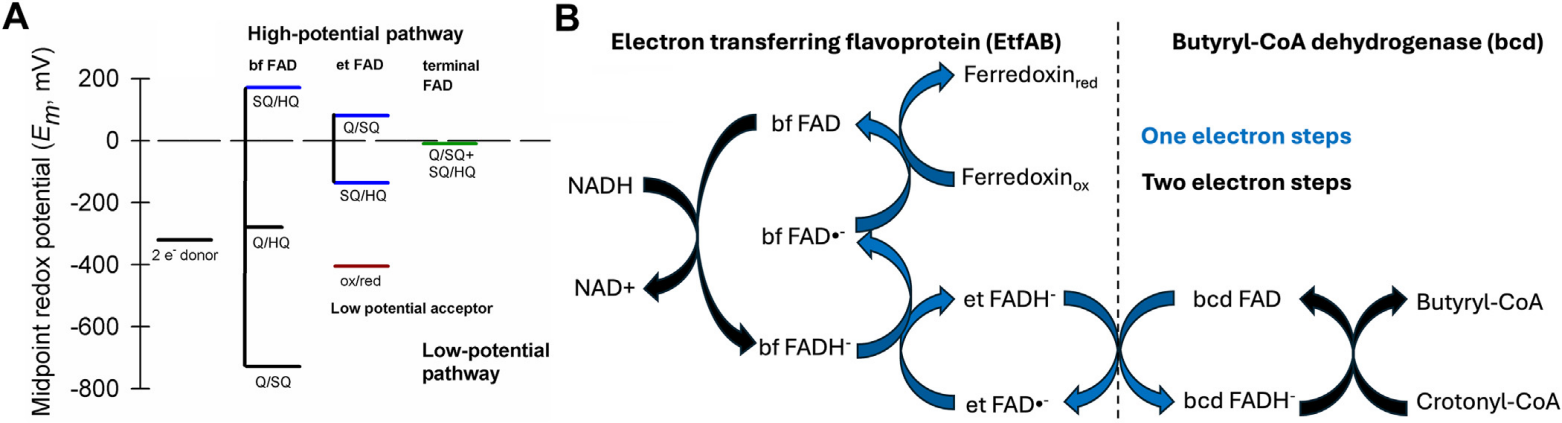
**Reduction potentials for a general flavin-based electron bifurcation system.***A*, the half-potentials of the flavins and electron donors and acceptors in a EtfAB-based system. The bf FAD on EtfAB has crossed half-potentials, meaning that the semiquinone state is highly unstable and has a low enough reduction potential to reduce ferredoxin. *B*, a schematic illustrating the path of electrons through the EtfAB:bcd system investigated here, starting with a median-potential two-electron donor, NADH, and passing through discrete high- and low-potential pathways to reduce separate high- and low-potential acceptors (crotonyl-CoA and ferredoxin, respectively).

A large family of flavin-based electron bifurcating systems possesses an electron-transferring flavoprotein (ETF) component (1). These bifurcating ETFs are closely related to but distinct from the non-bifurcating, canonical ETFs found in organisms in all three domains of life, including humans (3). Non-bifurcating ETFs contain one equivalent of FAD in the α subunit, and one of AMP in the β subunit; the lone FAD (the electron-transferring FAD, hereafter et FAD) has highly uncrossed potentials, with one-electron reduction yielding a very thermodynamically stable FAD•^-^. Bifurcating ETFs are also αβ dimers, with significant sequence and structural homology to the non-bifurcating ETFs, but the β subunits possess a second equivalent of FAD rather than AMP. It is this second FAD that is the site of electron bifurcation and is referred to as the bifurcating FAD (hereafter bf FAD).

The present work focuses on the crotonyl-CoA-dependent NADH:ferredoxin oxidoreductase EtfAB:bcd (for butyryl-CoA dehydrogenase)† from *Megasphaera elsdenii*, which has close homologs in *Clostridium difficile* and *Acidaminococcus fermentans*. The bcd component of the system has a third FAD (in addition to the two FADs present in the ETF), the bcd FAD, which is the site at which the high-potential acceptor crotonyl-CoA is reduced to butyryl-CoA. The product butyryl-CoA is an intermediate in reverse beta-oxidation and the synthesis of straight-chain fatty acids. The sequence of events in electron bifurcation in this system, hereafter referred to as EtfAB:bcd, is shown in Figure 1B and is as follows. NADH reduces the bf FAD *via* hydride transfer to the hydroquinone. A first, high-potential electron is transferred from the bf FADH^-^ to the et FAD (and ultimately on to the bcd FAD). The initial electron transfer out of the bf FADH^-^ leaves behind the strongly reducing bf FAD•^-^, which directly reduces ferredoxin. After reaction with a second equivalent of NADH (and reduction of a second equivalent of ferredoxin), a second high-potential electron is eventually delivered to the bcd FAD to reduce crotonyl-CoA to butyryl-CoA. As in all bifurcating systems that have been investigated, reduction of the high- and low-potential substrates is tightly coupled during steady-state turnover, with little or no “leakage” of the low-potential electron into the high-potential pathway despite the extreme thermodynamic favorability of the event. The tight coupling of reduction of the high- and low-potential acceptors is thus fundamentally kinetic in nature and appears to be an intrinsic property of the bifurcating FAD. This tight coupling does not appear to be strictly dependent on the simultaneous binding of low- and high-potential acceptors but is an intrinsic property of the bf FAD, as reflected by the observation that under at least certain conditions ferredoxin reduction can occur in these systems in the absence of the high-potential acceptor (4).

The X-ray crystal structure of EtfAB:bcd from *C. difficile* has been reported by Ermler and coworkers (5). The complex has a (bcd)_4_ core organized as a dimer of dimers, with each bcd subunit associated with an EtfAB dimer. The nearest approach of cofactors in neighboring EtfAB:bcd protomers (between bcd FADs) is > 35 Å, indicating that reducing equivalents are not transferred between protomers in the course of catalysis. In a given EtfAB:bcd protomer the et FAD lies within 8.4 Å of the bcd FAD, but 37 Å from the bf FAD. On the other hand, in the crystal structure of the isolated EtfAB from *A. fermentans* the et FAD lies approximately 14 Å from the bf FAD (5), and it is evident that the domain containing the et FAD is mobile, swinging through a 78-degree rigid-body rotation in the course of being reduced by the bf FAD and reoxidized by the bcd FAD. Interestingly, this same rotation is seen in simple ETFs as they interact with their physiological partners (generally ETF reductases and fatty acyl-CoA dehydrogenases) (6, 7), and appears to be a fundamental characteristic of electron transferring flavoproteins. The bcd component of the intact system, on the other hand, has essentially the identical structure to that seen for the isolated bcd from *M. elsdenii* (8), and as originally noted has significant structural homology to porcine medium chain acyl-CoA dehydrogenase (which accepts reducing equivalents from a simple, non-bifurcating ETF) (9).

We have previously examined the rapid-reaction kinetics of reduction of the *M. elsdenii* EtfAB by NADH (10), with a fast phase representing the initial reduction of the bf FAD that exhibits a hyperbolic dependence on [NADH] with a limiting k_red_ of 590 s^−1^ and a K_d_^NADH^ of 30 μM. This fast phase is then followed by a much slower and [NADH]-independent reduction of the et FAD to give fully reduced EtfAB with an observed rate constant of 2 s^−1^. Similarly, we have examined the reaction of reduced bcd with crotonyl-CoA (11), and find that the reaction occurs with a k_ox_ of 30 s^−1^ that is independent of [crotonyl-CoA], presumably due to an extremely low K_d_ for the high-potential substrate. We have also examined the rate of electron transfer from reduced EtfAB to oxidized bcd, and found that both one- and two-electron processes occur with a k_ET_ of 2 s^-1^. This rate constant is independent of protein concentration and is presumably due to electron transfer within the EtfAB:bcd complex, which forms rapidly and with a low K_d_. Having examined the kinetics of both isolated components of EtfAB:bcd, we examine here the rapid-reaction kinetics of the complete EtfAB:bcd system.

## Results

### The reduction of EtfAB:bcd with NADH

**Figure 2:**
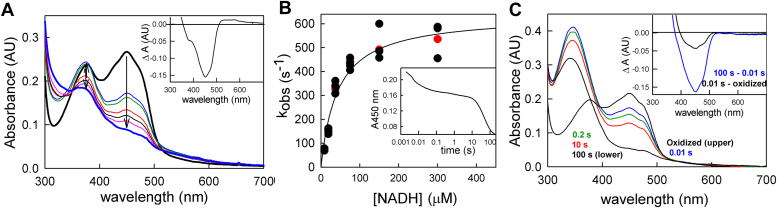
**The reaction of oxidized EtfAB:bcd complex with NADH.***A*, titration of 8 μM EtfAB:bcd with NADH, the upper blue represents the greatest accumulation of both anionic semiquinone and neutral semiquinone. Inset, the difference spectrum between the oxidized (*bold black*) and reduced (*bold blue*) shows the total absorbance change in the titration; negative features indicate loss while positive features represent accumulation. *B*, the reaction of 6.5 μM EtfAB:bcd with various concentrations of NADH; the apparent k_red_ is 640 s^−1^ and the apparent K_d_ is 43 μM. The red points are averages of the raw data shown as black points. Inset, a representative kinetic transient at 37 μM NADH at 450 nm showing that the initial reduction of the bf FAD and the reduction of the complete system are separated temporally. *C*, rapid-reaction kinetics seen in the course of the reaction of complex with 37 μM NADH, showing the eventual complete reduction of the system and the small accumulation and subsequent loss of anionic semiquinone demonstrated by the shift of the short wavelength maxima from 377 nm to 340 nm. Inset, difference spectra at 37 μM NADH, showing the rapid reduction of the bf FAD (first 0.01 s, black) and the reduction of all three flavins (total absorbance, blue); negative features indicate loss while positive features represent accumulation. Both experiments were performed in 50 mM Tris HCl, 150 mM NaCl, pH 7.5. The titration was performed at 25 °C while the stopped-flow experiments at 10 °C.

We have shown in previous work that FAD•^-^ accumulates transiently in the course of reductive titrations of EtfAB with NADH and that the two-electron reduced EtfAB, EtfAB_2e−_, that is formed initially in the course of the reaction appears to exist in a conformational equilibrium between two states, one in a “bifurcation-ready” configuration with the bf FAD fully reduced, and a second in which the half-potentials of the bf FAD have become uncrossed, resulting in a distribution of the two electrons between the bf FAD and et FAD such that both are in the FAD•^-^ state (10). In the present work, we first performed a reductive titration of the intact EtfAB:bcd complex (formed simply by mixing equimolar concentrations of the two separately expressed and purified recombinant components) with NADH. As shown in Figure 2*A*, both FADH• and FAD•^-^ do indeed appear transiently in the course of the titration, as reflected in the simultaneous transient absorption increases at ∼550 and ∼377 nm, respectively. The maximum centered around 550 nm and the ratio of the 377 nm and 450 nm minima in Figure 2*A* inset, imply the presence of both semiquinones at the end of titration as well. The accumulation of semiquinone results in an increase of absorbance of 377 nm, which increases the ratio between the absorbances of 377 nm to 450 nm. The observed accumulation of semiquinone seen with the EtfAB:bcd complex is consistent with that observed with the isolated EtfAB and bcd components, and indicates that the half-potentials of a portion of the bf FAD again become uncrossed in EtfAB:bcd_2e_-, just as seen in EtfAB_2e_-. Confirmation of the uncrossing of the bf FAD at short times is confirmed by EPR (Fig. S1).

As previously reported, the rapid-reaction kinetics for the reduction of isolated EtfAB by NADH yield biphasic kinetics (10). The fast phase, accounting for approximately 50% of the total absorbance change, exhibits a spectral change consistent with the initial rapid reaction of the bf FAD to the hydroquinone, with a hyperbolic dependence of the observed rate constant on [NADH], that yields a limiting k_red_ of 590 s^−1^ and an apparent K_d_ of 30 μM (10). The inclusion of the bcd component to form the full complex does not significantly change the kinetics of the fast phase of the reaction, with an apparent k_red_ of 640 s^−1^ and apparent K_d_ of 43 μM (Fig. 2*B*). This initial kinetic phase accounts for only approximately one-third of the total absorbance change (Fig. 2*B*, inset), however, consistent with the initial reduction of only one of the three flavins present in the complex, the bf FAD. The remaining two-thirds of the absorbance change occurs on a much slower time scale and reflects the transfer of reducing equivalents out of the bf FADH^-^ to the et and bcd FADs (Fig. 2*B* inset), with a rate that is independent of [NADH] but modestly dependent on the concentration of EtfAB:bcd, suggesting that it involves intermolecular electron transfer of transiently formed bf FAD•^-^ into the high-potential pathway of another molecule of the complex. Figure 2*C* shows the spectra seen in the course of the reaction with the spectral changes associated with the two observed kinetic phases shown in the inset.

### The reaction of fully reduced EtfAB:bcd with crotonyl-CoA

**Figure 3:**
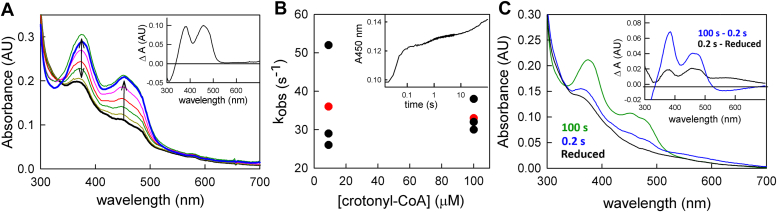
**The reaction of fully reduced EtfAB:bcd with crotonyl-CoA.***A*, oxidative titration of 11 μM reduced EtfAB:bcd in the absence of ferredoxin. There is an accumulation of anionic semiquinone throughout the titration. Inset, difference spectrum showing the total change throughout the titration; negative features indicate loss while positive features represent accumulation. *B*, the reaction of fully reduced EtfAB:bcd with crotonyl-CoA. The apparent k_ox_ is ∼ 30 s^−1^ and there is no dependence on [crotonyl-CoA] as shown by the red points representing the average rates. Inset, transient showing the temporal separation between the reoxidation of the bcd FAD and the other FADs from EtfAB. *C*, rapid-reaction kinetics of 5 μM of pre-reduced EtfAB:bcd_6e_-reacted with crotonyl-CoA: black, reduced EtfAB:bcd before reaction with crotonyl-CoA; blue, 0.2 s after mixing with crotonyl-CoA; green, the endpoint of the reaction. Inset, difference spectra of the reaction: black, the difference spectrum of 0.2 s minus reduced; blue, the difference spectrum of 100 s minus 0.2 s; negative features indicate loss while positive features represent accumulation. The absorbance at 377 nm is due to the anionic semiquinone while the long-wavelength absorbance is due to the charge-transfer complex of the reduced bcd and crotonyl-CoA. Both experiments were performed in 50 mM Tris HCl, 150 mM NaCl, pH 7.5. The titration was performed at 25 °C while the stopped flow experiments at 10 °C.

We next prepared the fully reduced EtfAB:bcd (prepared by titration with sodium dithionite to yield EtfAB:bcd_6e−_) and titrated it with the high-potential acceptor crotonyl-CoA in the absence of the low potential acceptor ferredoxin. As shown in Figure 3*A*, the observed spectral changes reflect a significant accumulation of anionic semiquinone in the course of the titration. This may be due either to the et FAD or bcd FAD, as we have previously shown that the bcd semiquinone ionizes when a CoA substrate (crotonyl-CoA or butyryl-CoA) or analog (acetoacetyl-CoA) binds (11). It is noteworthy that the EtfAB:bcd complex is not fully reoxidized at the end of the titration even in the presence of excess crotonyl-CoA, as reflected in both the increase in absorbance at 377 nm during the course of titration and the ratio of absorption at 377 nm to 450 nm in the difference spectrum (compare Figure 2, Figure 3*A* and 3*A*). This incomplete reoxidation appears to be a consequence of two things. First, in the absence of the low-potential acceptor, ferredoxin, the first electron transfer out of the bf FADH^-^ into the high-potential pathway (accompanied by the generation of the low-potential electron) may only occur extremely slowly to fully oxidize the bf FAD and allow reaction with a second (and eventually third) equivalent of crotonyl-CoA (a leakage that may indeed involve intermolecular rather than intramolecular electron transfer, which we have shown to occur on a tens of minutes time scale). Second, it may be that in the configuration in which the half-potentials of the bf FAD have become uncrossed further reoxidation by crotonyl-CoA becomes unfavorable.

Figure 3*B* shows the rapid-reaction kinetics of reoxidation of dithionite-reduced EtfAB:bcd_6e_- by crotonyl-CoA, which exhibits a [crotonyl-CoA]-independent rate constant of 30 s^-1^. Although the lower concentration of crotonyl-CoA used is only equimolar with the EtfAB:bcd concentration used in the experiment, the observed kinetics are exponential rather than second-order, consistent with the very tight binding as reflected in the substrate-independent rate constant. The observed rate constant agrees very well with our previous results using the isolated bcd, which yielded exponential transients and a similarly [crotonyl-CoA]-independent rate constant of 30 s^-1^ over a much larger range of crotonyl-CoA concentrations (11). Also consistent with previous results with the isolated bcd, in the presence of crotonyl-CoA, the semiquinone accumulating on the bcd FAD is the anionic rather than neutral form, the long-wavelength absorbance accumulating transiently being due to a charge-transfer complex rather than a neutral bcd semiquinone (Fig. 3*C* inset); these complexes are between bcd_ox_:butyryl-CoA and bcd_red_:crotonyl-CoA (11). The 450 nm transient in Figure 3*B* inset illustrates 3 distinct processes. The first, constitutes the reoxidation of reduced bcd by crotonyl-CoA occurring at the rate of 30 s^−1^ ending at approximately 0.2 s as indicated above. The second phase from 0.2 to 10 s, can be characterized by an exponential process at 1.5 s^−1^, which is consistent with the rate of intramolecular ET from reduced EtfAB to bcd as previously described (11). The last phase from 10 s to the end of the reaction is indicative of intermolecular electron transfer as it is not well-resolved kinetically and occurs on the tens of seconds time scale. The difference spectra in Figure 3*C* inset indicate the reoxidation of bcd (black) in the first phase as expected and the contribution of both phases on the longer time scale resulting in primarily semiquinone accumulation (blue) as both the intra- and intermolecular ET is predominately one-electron.

### The reaction of EtfAB:bcd_6e_-with crotonyl-CoA in the presence of ferredoxin

**Figure 4:**
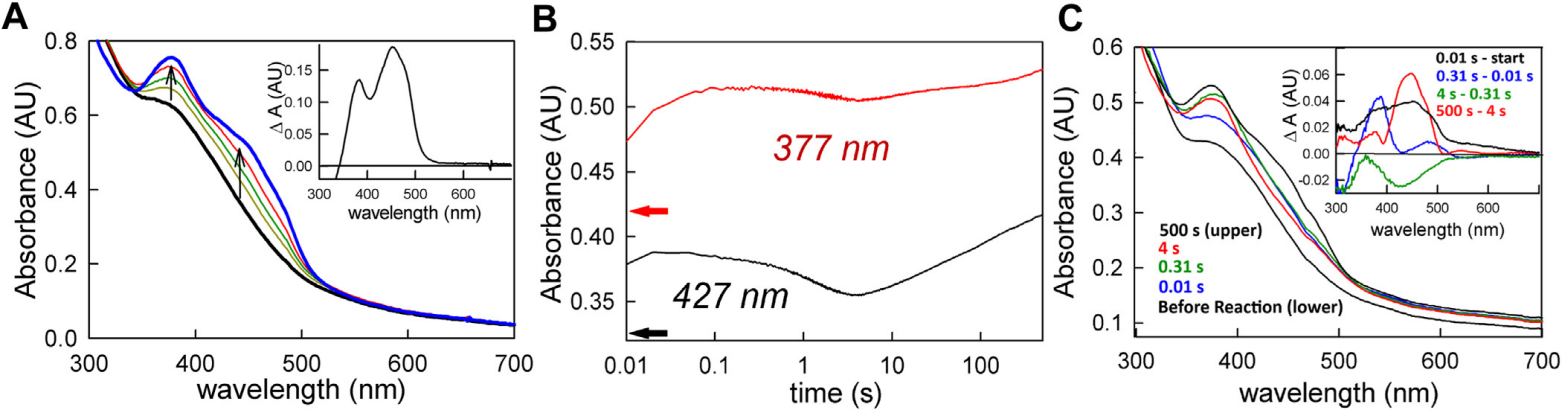
**The reaction of fully reduced EtfAB:bcd with crotonyl-CoA in the presence of ferredoxin.***A*, titration of 11 μM EtfAB:bcd with crotonyl-CoA in the presence of 20 μM ferredoxin: The bold black spectrum shows the fully reduced EtfAB:bcd alongside oxidized ferredoxin, and bold blue spectrum shows the fully oxidized EtfAB:bcd with excess crotonyl-CoA, and oxidized ferredoxin. Inset, difference spectrum showing the total change throughout the titration; negative features indicate loss while positive features represent accumulation. *B*, kinetic transients at 427 nm (*black*) showing the extent of ferredoxin reduction, and at 377 nm (*red*) showing semiquinone accumulation when 4 μM prereduced EtfAB:bcd is reacted with 15 μM ferredoxin and 50 μM crotonyl-CoA. The red and black arrows correspond to the starting points of each transient, before mixing. *C*, rapid-reaction kinetics of 4 μM prereduced EtfAB:bcd reacted with 15 μM ferredoxin and 50 μM crotonyl-CoA: before the reaction (*black*), 0.01 s into the reaction (*blue*), after 0.31 s (*green*), after 4 s (*red*), and after 500 s (*upper black*). Inset, difference spectra showing the spectral changes associated with the reoxidation of bcd by crotonyl-CoA (0.01 s minus start, *black*), the approach to ferredoxin reduction (0.31 s minus 0.1 s, *blue*), ferredoxin reduction (4 s minus 0.31 s, *green*), and the eventual reoxidation of the system (500 s minus 4 s, *red*); negative features indicate loss while positive features represent accumulation. Experiments were performed in 50 mM Tris HCl, 150 mM NaCl, pH 7.5, 25 °C.

Significantly, when the equilibrium titration of EtfAB:bcd_6e_-with crotonyl-CoA is repeated in the presence of excess ferredoxin (Fig. 4*A*), more complete reoxidation is observed than in the absence of the low-potential acceptor (compare the relative ratio of the 377 and 450 nm peaks in the oxidized-minus-reduced difference spectrum in Figure 4*A* inset to that in Fig. 3*A*, and also the total absorbance changes at 450 nm in the presence and absence of the ferredoxin in the two experiments). With ferredoxin present, the bf FAD•^-^ generated after a first (or second) successful bifurcation event occurring in the course of reoxidation, reacts rapidly with the ferredoxin present and results in the full reoxidation of the bf FAD. Interestingly, while the observed difference spectrum (Fig. 4*A* inset) reflects essentially complete reoxidation of EtfAB:bcd, there is no contribution of the expected spectral change due to reduction of ferredoxin, indicating that it had become reoxidized in the course of the titration by reducing another molecule of EtfAB:bcd (presumably into its et and/or bcd FADs, see the kinetics below).

The kinetics of the reoxidation of EtfAB:bcd_6e_- by crotonyl-CoA are also affected by excess ferredoxin. Tracking ferredoxin reduction at 427 nm, (Fig. 4*B*, black transient), and the initial accumulation of FAD•^-^ at 377 nm (Fig. 4*B*, red transient), the magnitude of the absorbance change reflects the reduction of one equivalent of ferredoxin per EtfAB:bcd; it is evident that the observed kinetics are again complex. Absorbance spectra seen at specific points in the course of the reaction are shown in Figure 4*C*, with difference spectra for each kinetic phase in the inset. The spectral change associated with the initial phase of the reaction (Fig. 4*C*, inset, black spectrum) is consistent with this process involving the rapid two-electron reoxidation of bcd by crotonyl-CoA. The second phase of the reaction exhibits a spectral change (Fig. 4*C*, inset, blue spectrum) reflecting electron transfer from the et FADH^-^ to the now reoxidized bcd FAD, both of which become FAD•^-^. The spectral change associated the third phase reflects ferredoxin reduction as a result of bifurcation at the bf FAD concomitant with reduction of the et FAD•^-^ to the hydroquinone, and possibly further electron transfer on to the bcd flavin (Fig. 4*C*, inset, green spectrum). Based on the amplitude of the absorbance change at 377 nm, we estimate that ∼15% of the complex is in this bifurcating state at any given time. The spectral change associated with the final process (Fig. 4*C*, inset, red spectrum) involves full oxidation of the et and bcd FADs due to the presence of excess crotonyl-CoA. This last spectral change also includes a contribution due to reoxidation of the now-reduced ferredoxin, consistent with the results of the titration shown in Figure 4*A* indicating that it had indeed become reoxidized in the course of the reaction.

We did not examine the reaction of EtfAB:bcd_6e_- with ferredoxin in the absence of crotonyl-CoA. With the et and bcd FADs fully reduced at the outset of the experiment, there is no way for the fully reduced bf FADH^-^ to bifurcate and transfer a first, high-potential electron into the high-potential pathway to generate the low-potential bf FAD•^-^ as both et and bcd FADs are already fully reduced. Instead, the enzyme-monitored turnover experiments described in the next section were performed.

### Enzyme-monitored turnover with EtfAB:bcd_ox_

**Figure 5:**
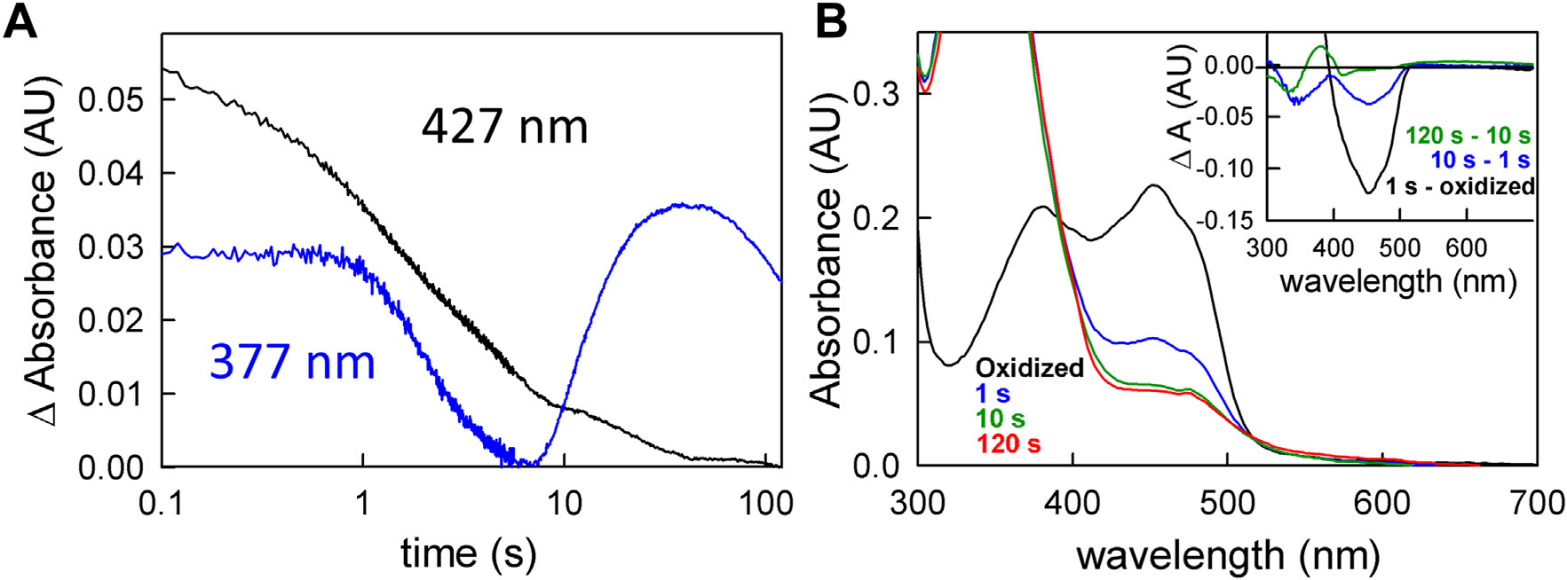
**Crotonyl-to butyryl-CoA turnover with EtfAB:bcd**_**ox**_**and excess NADH.***A*, the representative transients at 427 nm (*black*) and 377 nm (*blue*) of the reaction of 7 μM fully oxidized EtfAB:bcd complex with 90 μM NADH and 50 μM crotonyl-CoA shown to 120 s. *B*, rapid-reaction kinetics for the reaction of 7 μM oxidized EtfAB:bcd (*black*) with 90 μM NADH and 50 μM crotonyl-CoA. Inset, the difference spectra showing 1 s minus oxidized (*black*), 10 s minus 1 s (*blue*), and 120 s minus 10 s (green); negative features indicate loss while positive features represent accumulation. Experiments were performed in 50 mM Tris HCl, 150 mM NaCl, pH 7.5, 25 °C.

The steady-state kinetic behavior of EtfAB:bcd has been previously described (12), but enzyme-monitored turnover experiments, in which spectral changes directly attributable to the enzyme complex are monitored in the course of reaction with all three substrates, are required to fully reconcile the rapid-reaction kinetics with catalytic turnover. As a preliminary, we performed a partial enzyme-monitored turnover experiment in the absence of ferredoxin, where we mixed oxidized EtfAB:bcd, EtfAB:bcd_ox_, with solutions containing NADH and crotonyl-CoA, to mimic conditions in the above experiments done in the presence of ferredoxin. Following the absorbance change at 427 nm (Fig. 5*A*, black transient), continual flavin reduction throughout the course of the reaction is observed. The transient can be divided into three phases. An initial dead-time spectral change involves the rapid reduction of the bf FAD by NADH as well as some electron transfer into the et FAD, although we note that given the large excess of NADH used, the formation of anionic semiquinone, giving an absorbance increase at 377 nm (blue transient) is largely obscured by the larger absorbance decreases associated with both the oxidation of NADH and reduction of the bf FAD during this portion of the reaction. A second phase occurs until about 10 s, consisting of reduction of the et FAD (Fig. 5*B* inset, blue spectrum). On a much longer time scale of 10 to 50 s, however, FAD•^-^ does accumulate, as reflected by the absorbance increase at 377 nm in the blue transient of Figure 5*A* and the positive 377 nm maxima in the green difference spectrum in the inset to Figure 5*B*. This is again a reflection of the uncrossing of the bf FAD half-potentials in a subset of the EtfAB:bcd population. The important implication is that the uncrossing of the bf FAD half-potentials previously observed with the isolated EtfAB is also seen in the EtfAB:bcd complex. This is consistent with not only the titration behavior described above, but also the rapid-reaction kinetics of both the isolated reduction of EtfAB and the reoxidation of bcd as reported previously (10, 13).

**Figure 6:**
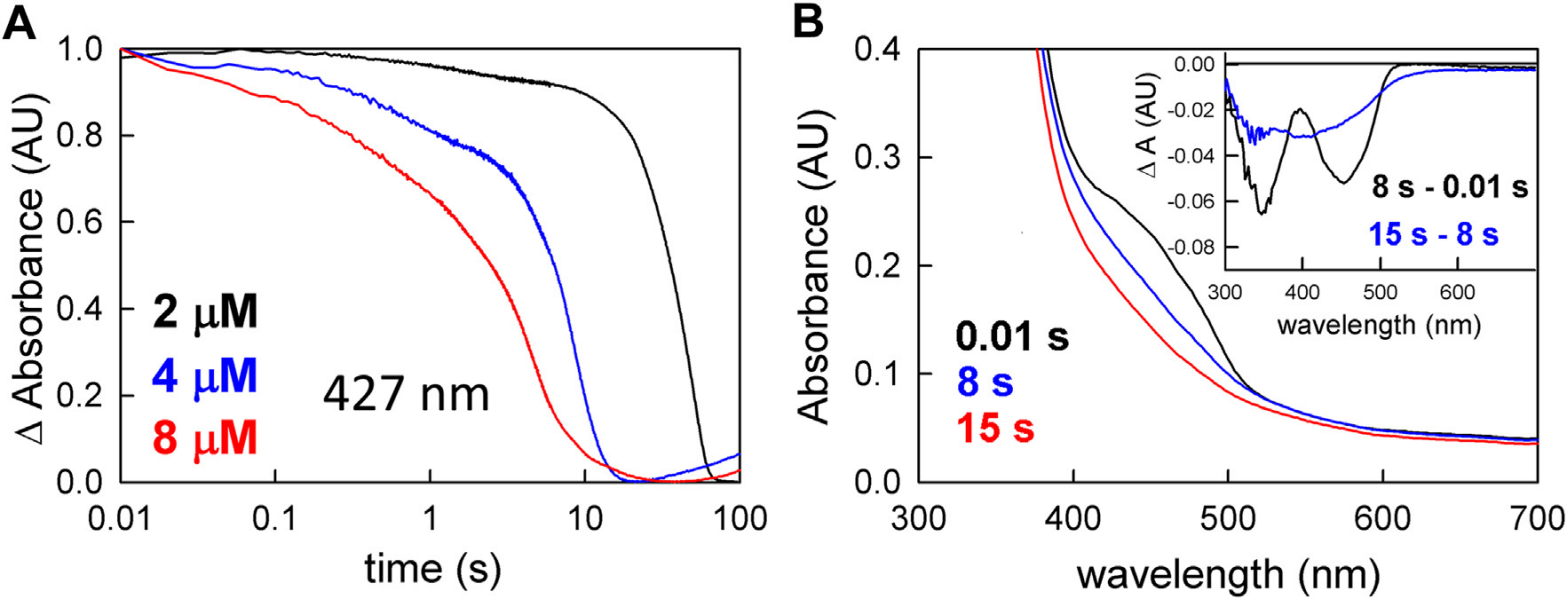
**Enzyme monitored turnover of ferredoxin reduction with increasing [EtfAB:bcd]**_**ox**_**.***A*, 427 nm transients used to determine ferredoxin reduction upon the reaction of varying concentrations of EtfAB:bcd (2 μM (*black*), 4 μM (*blue*), and 8 μM (*red*)) with a solution of 90 μM NADH, 50 μM crotonyl-CoA, and 15 μM ferredoxin. The transients were normalized to the total ΔA to emphasize the separation between the approach to steady-state and reduction phases at the lowest concentration of the complex used. *B*, rapid-reaction kinetics for the reaction with 4 μM EtfAB:bcd indicating the endpoints of the distinct phases of reduction: first observed spectrum at 0.01 s (*black*), end of flavin reduction at 8 s (*blue*) and the end of ferredoxin reduction at 15 s (*red*). Inset, the difference spectra of 8 s minus 0.01 s showing flavin reduction with 450 nm maxima and 15 s minus 8 s showing predominately ferredoxin reduction with 420 nm maxima; negative features indicate loss while positive features represent accumulation. Experiments were performed in 50 mM Tris HCl, 150 mM NaCl, pH 7.5, 25 °C.

For full enzyme-monitored turnover experiments, where ferredoxin is included as well as NADH and crotonyl-CoA, account must be taken of the absorbance change at 427 nm due to ferredoxin reduction (Δε of 8.3 mM^−1^ cm^−1^ (3)). As shown in Figure 6*A*, on mixing oxidized EtfAB:bcd with a mixture of NADH, crotonyl-CoA and ferredoxin an approach to the steady-state occurs in an [EtfAB:bcd]-dependent manner over the first 2 to 20 s of the reaction. The spectral change associated with this approach to steady-state reflects flavin reduction of one of the three flavins to the hydroquinone, undoubtedly the bf FAD, with characteristic negative features at 450 and 360 nm (Fig. 6*B*, inset, black difference spectrum). There is no significant positive feature at 377 nm in this difference spectrum, as oxidation of NADH (shown as a minimum at ∼350 nm) obscures any accumulation of FAD•^-^. Importantly, no ferredoxin reduction is evident in this approach to the steady-state phase of the reaction. The system next enters a linear steady-state phase at ∼8 s that involves extensive ferredoxin reduction, as reflected in the broad absorption minimum centered at ∼405 nm in the difference spectrum associated with this phase (Fig. 6*B*, inset, blue difference spectrum). There is little flavin contribution to this spectral change, indicating that the overall level of EtfAB:bcd reduction does not change significantly throughout the steady-state, particularly with regard to the level of FAD•^-^ (which would be manifested as a significant absorbance change in the 377 nm region in the blue difference spectrum, which is not observed). The rate of ferredoxin reduction in this steady-state phase is 0.2 s^-1^, consistent with previous steady-state studies (12). The steady-state phase is followed by a final phase representing reduction of EtfAB:bcd by the excess NADH present upon depletion of the limiting ferredoxin in the reaction mix.

**Figure 7:**
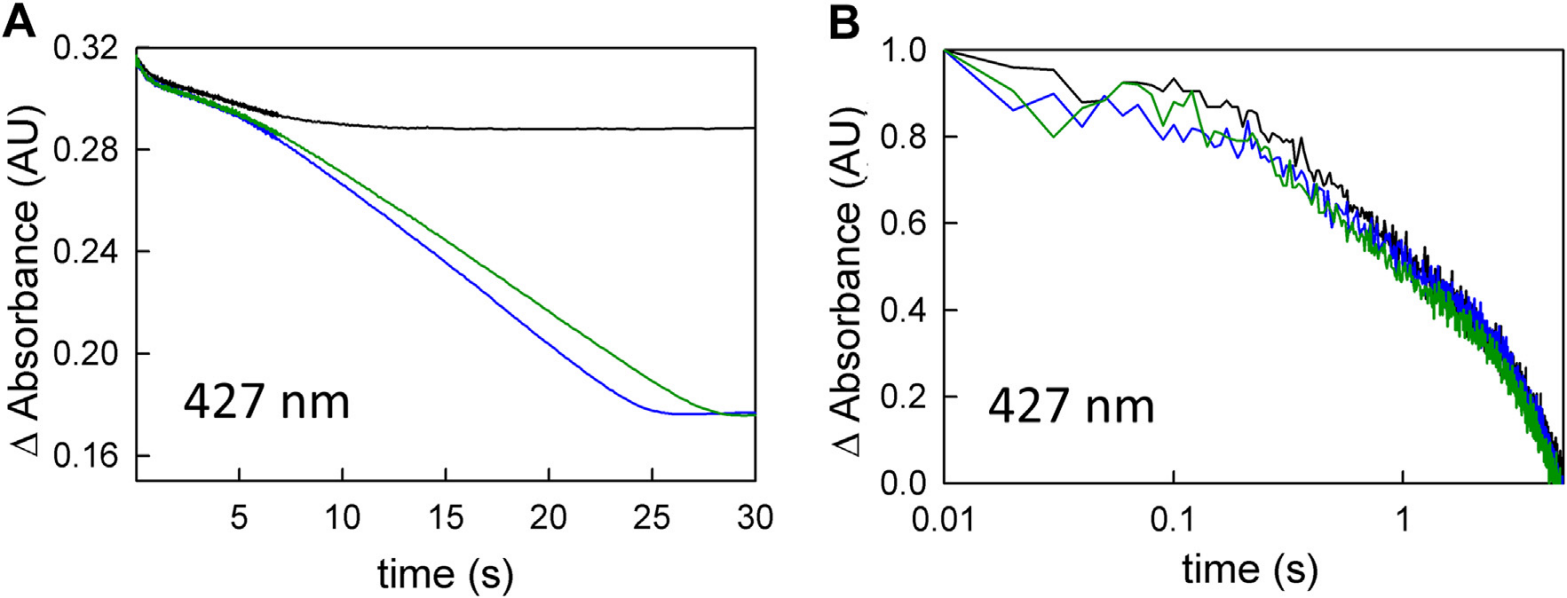
**Enzyme monitored turnover with varying crotonyl-CoA concentrations.***A*, kinetic transients monitored at 427 nm of the enzyme-monitored turnover experiments when 5 μM EtfAB:bcd, 15 μM ferredoxin, and 90 μM NADH are reacted with crotonyl-CoA concentrations of 5, 50, and 150 μM (*black*, *blue*, *green*, respectively). *B*, the expansion of the kinetic transients over the first 5 seconds of reaction, normalized to demonstrate that the approach to steady-state is essentially independent of [crotonyl-CoA]. Experiments were performed in 50 mM Tris HCl, 150 mM NaCl, pH 7.5, 25 °C.

We have also examined the effect of changing the concentration of the high potential acceptor, crotonyl-CoA, on the kinetics of the enzyme-monitored turnover experiment, following the reaction again at 427 nm, using 5, 50, and 150 μM crotonyl-CoA, ranging from stoichiometric to a pseudo-first-order excess relative to EtfAB:bcd. As shown in Figure 7*A*, the higher concentrations of crotonyl-CoA (50 and 150 μM) exhibit comparable rates and amplitudes during the ferredoxin reduction phase of the reaction. At 5 μM concentration of crotonyl-CoA, on the other hand, a much smaller amplitude is observed due to the limiting, low crotonyl-CoA concentration. As a result, the amplitude change due to ferredoxin reduction is low and swamped by the larger spectral changes due to the flavins of EtfAB:bcd. On the other hand, the initial approach to steady-state is essentially independent of the crotonyl-CoA concentration, as shown in the three normalized transients in Figure 7*B*.

### Enzyme-monitored turnover with EtfAB:bcd_1e_-

It has been suggested that the EtfAB:bcd complex must be reductively primed for bifurcation (5), with the et FAD operating principally if not exclusively through the SQ/HQ one electron couple in shuttling high-potential electrons from the bf FAD to the bcd FAD. It is not straightforward to establish that this is a requirement for bifurcation, however, as it is to be expected that the three-FAD system would become partially reduced in the course of turnover in any case (non-bifurcating enzymes with multiple redox-active centers such as xanthine oxidase become partially reduced in the course of turnover, for example (14)). To address the issue in the case of EtfAB:bcd, we have repeated the above work starting with EtfAB:bcd_1e_-, where the single reducing equivalent resides on the et FAD•^-^. Reduction of the et FAD was accomplished by taking advantage of its uniquely photoactive nature (15) as demonstrated in Fig. S2, following its photoreduction spectrophotometrically. The et FAD•^-^ thus generated was stable and the electron did not transfer to the oxidized bcd FAD when the EtfAB_1e_-was mixed with bcd (which would have resulted in loss of et FAD•^-^ and formation of bcd FADH• which was not observed, data not shown), so the photoreduced EtfAB_1e-_ and bcd could be precomplexed without difficulty.

**Figure 8:**
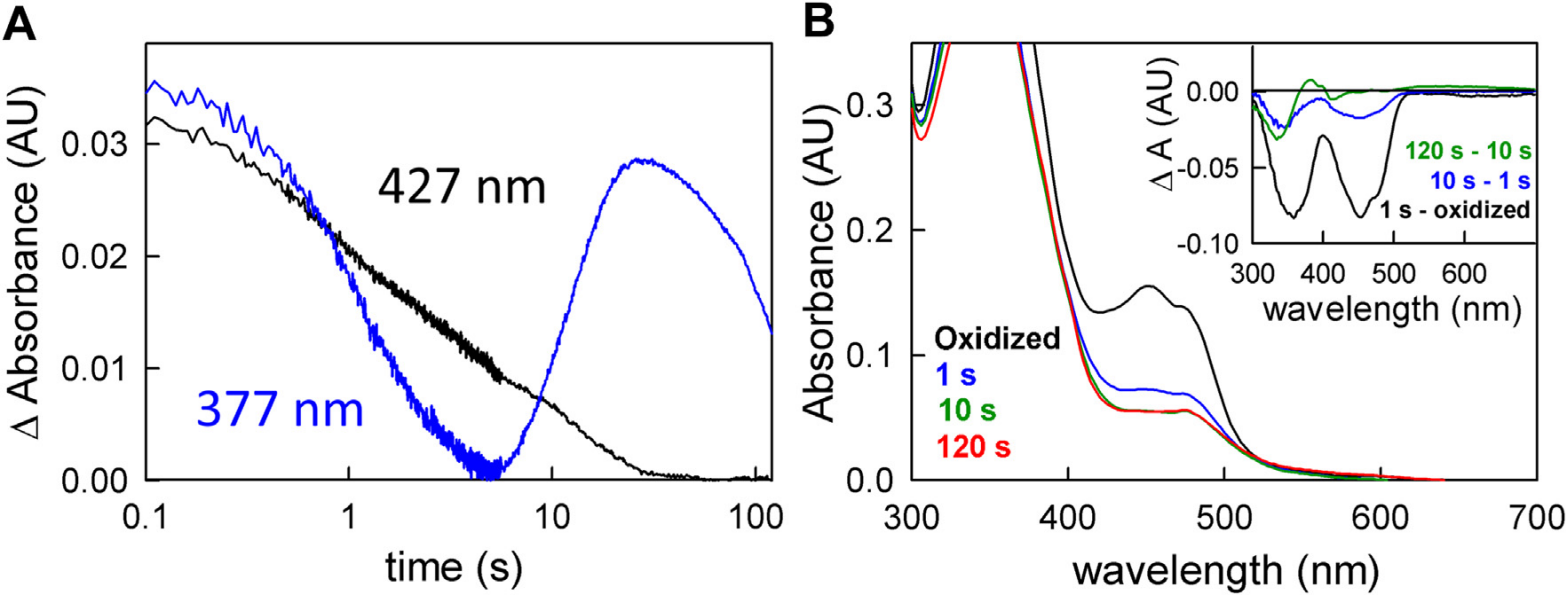
**Crotonyl-to butyryl-CoA turnover via reaction of excess NADH with EtfAB:bcd**_**1e-**_**.***A*, the representative transients at 427 nm (black) and 377 nm (*blue*) of the reaction of 7 μM one-electron reduced EtfAB:bcd complex with 90 μM NADH and 50 μM crotonyl-CoA shown to 120 s. *B*, rapid-reaction kinetics for the 7 μM one-electron reduced EtfAB:bcd (*black*) reacted with 90 μM NADH and 50 μM crotonyl-CoA. Inset, the difference spectra showing 1 s minus oxidized (*black*), 10 s minus 1 s (*blue*), and 120 s minus 10 s (*green*); negative features indicate loss while positive features represent accumulation. Experiments were performed in 50 mM Tris HCl, 150 mM NaCl, pH 7.5, 25 °C.

Figure 8 shows the same experiment as depicted in Figure 5, but reacting EtfAB:bcd_1e_- (rather than EtfAB:bcd_ox_) with NADH and crotonyl-CoA. The same kinetic phases seen with the oxidized enzyme above are seen with EtfAB:bcd_1e_-, with an initial reduction of the bf FAD by NADH, followed by electron transfer to the subsequent FADs, and then accumulation of anionic semiquinone at longer timescales. Again, the accumulation of semiquinone is reflected in the increase of absorbance at 377 nm, albeit to a lesser extent compared to the oxidized complex, as there is already et FAD•^-^ present in the one-electron reduced complex. As reflected in the spectral changes shown in both Figures 5*B* and 8*B*, the complexes equilibrate to forms with mostly hydroquinone and some semiquinone (*e.g.*, Fig. 8*B*, red spectrum), demonstrating that some flavin reduction occurs in the presence of both reducing and oxidizing substrates, a point that could be catalytically relevant (see below).

**Figure 9:**
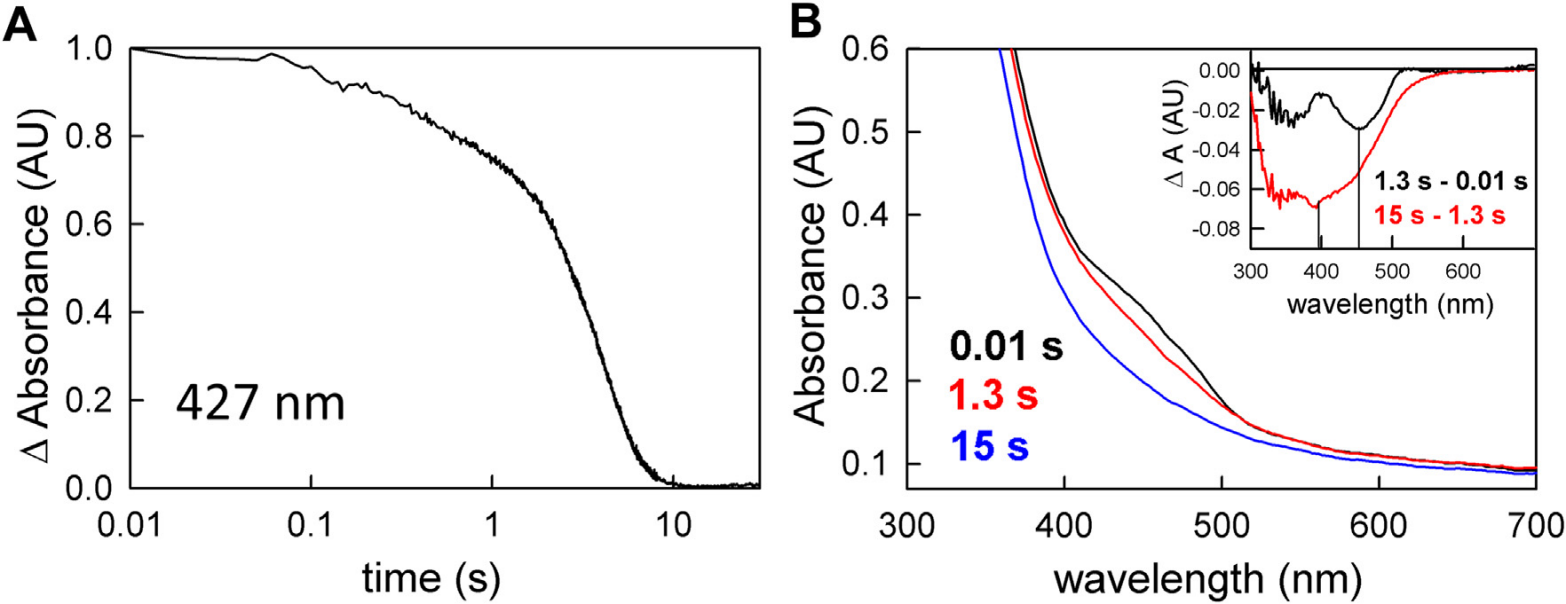
**Enzyme-monitored turnover with EtfAB:bcd**_**1e**_**-.***A*, 427 nm transient (normalized to the total ΔA) used to determine ferredoxin reduction upon reaction of 4 μM EtfAB:bcd_1e_-with 120 μM NADH, 50 μM crotonyl-CoA, and 14 μM ferredoxin. *B*, rapid-reaction kinetics showing the spectral changes for the different phases of the reaction: the first observed spectrum at 0.01 s (*black*), the end of flavin reduction at 1.3 s (*red*), and the end of ferredoxin reduction at 15 s (*blue*). Inset, difference spectra showing the extent of flavin reduction (black, 1.3 s minus 0.01 s) with a minimum at 450 nm, and ferredoxin reduction (*red*, 15 s minus 1.3 s) with a minimum at ∼400 nm; negative features indicate loss while positive features represent accumulation. Experiments were performed in 50 mM Tris HCl, 150 mM NaCl, pH 7.5, 25 °C.

The results of the full enzyme-monitored turnover experiment, including ferredoxin with NADH and crotonyl-CoA, are shown in Figure 9. Figure 9*A* shows that the approach to steady-state phase, when EtfAB:bcd_1e-_ is mixed with all three substrates, is shortened by roughly fivefold compared to that seen with the fully oxidized complex, while the steady-state involving ferredoxin reduction remains essentially unchanged, at 0.3 s^−1^. The decrease in the length of the approach to steady-state phase supports the hypothesis that the et FAD must be reduced to the FAD•^-^ state before the onset of bifurcation and ferredoxin reduction. The spectral change associated with the approach to steady-state phase with EtfAB:bcd_1e_- has changed as well: instead of an accumulation of anionic semiquinone (as seen when starting with EtfAB:bcd_ox_), the difference spectrum reflects reduction of oxidized flavin to the hydroquinone state (Fig. 9*B* inset, black difference spectrum). The absence of semiquinone accumulation in the approach to steady-state indicates the et FAD•^-^ is indeed the preferred oxidation state when the protein is turning over and bifurcating. Further, the inability of the electron on the et FAD•^-^ to be transferred to the bcd supports the notion that the et FAD operates between the SQ/HQ couple. As with EtfAB:bcd_ox_, the spectral change associated with the steady-state phase of the reaction reflects reduction of ferredoxin with little change in the overall level of reduction of the complex’s flavins.

### Enzyme-monitored turnover with post-catalytic EtfAB:bcd

**Figure 10:**
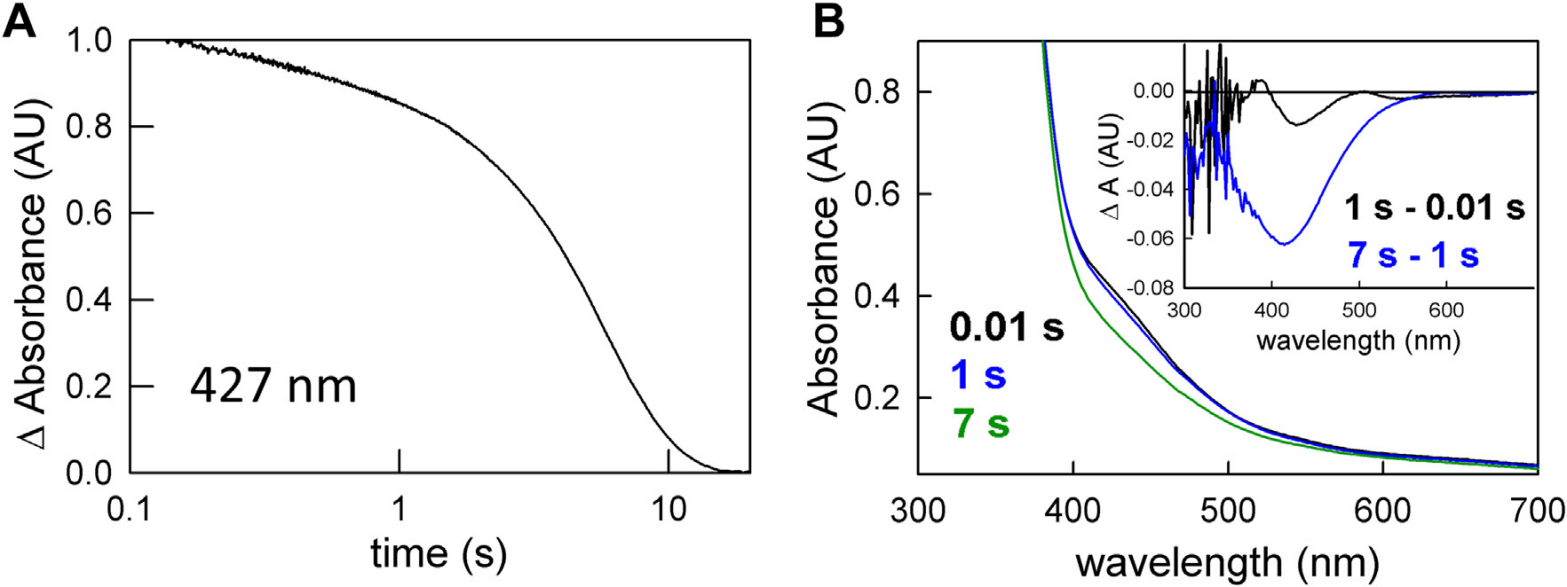
**Enzyme monitored turnover with post-catalytic EtfAB:bcd.***A*, 427 nm transient (normalized to the total ΔA) of 5 μM EtfAB:bcd first reacted with 90 μM NADH and 50 μM crotonyl-CoA and subsequently reacted with 90 μM NADH, 50 μM crotonyl-CoA and 15 μM ferredoxin. *B*, rapid-reaction kinetics showing the spectral changes for the different phases of the reaction: the first observed spectrum at 0.01 s (*black*), the end of the approach phase at 1 s (*blue*), and the end of ferredoxin reduction at 7 s (*green*). Inset, the difference spectrum of 1 s minus 0.01 s (*black*) shows reduced ferredoxin and a small amount of semiquinone formation as well as pure ferredoxin reduction for the difference spectrum at 7 s minus 1 s; negative features indicate loss while positive features represent accumulation. Experiments were performed in 50 mM Tris HCl, 150 mM NaCl, pH 7.5, 25 °C.

While the duration of the approach to a steady-state phase of the enzyme-monitored turnover experiment is significantly shortened by pre-reduction of the et FAD to the semiquinone, it is not abolished. We were thus interested to know whether EtfAB:bcd that had previously undergone turnover behaved differently than the fully oxidized complex. The experiment involves a reaction of EtfAB:bcd (5 μM) that had previously been exposed to excess NADH (90 μM) and limiting crotonyl-CoA (50 μM) but not ferredoxin to generate a complex that is predominately fully reduced, but containing some anionic semiquinone (as seen in Fig. 5*B*). When reacting this post-catalytic EtfAB:bcd with ferredoxin (15 μM), as well as more NADH (90 μM) and crotonyl-CoA (50 μM), the approach to steady-state in the enzyme-monitored turnover experiment is even shorter than seen with EtfAB:bcd_1e_-. More importantly, the spectral change associated with this phase of the reaction reflects not only flavin reduction but also significant ferredoxin reduction (Fig. 10*B* inset). The steady-state phase is now solely due to ferredoxin reduction, and with the same k_cat_ of 0.2 s^-1^. In comparing the behavior of EtfAB:bcd_1e_- and the post-catalytic complex, the significant conclusion is that reduction of the et FAD to the semiquinone is necessary but not sufficient for bifurcation and ferredoxin reduction.

**Figure 11:**
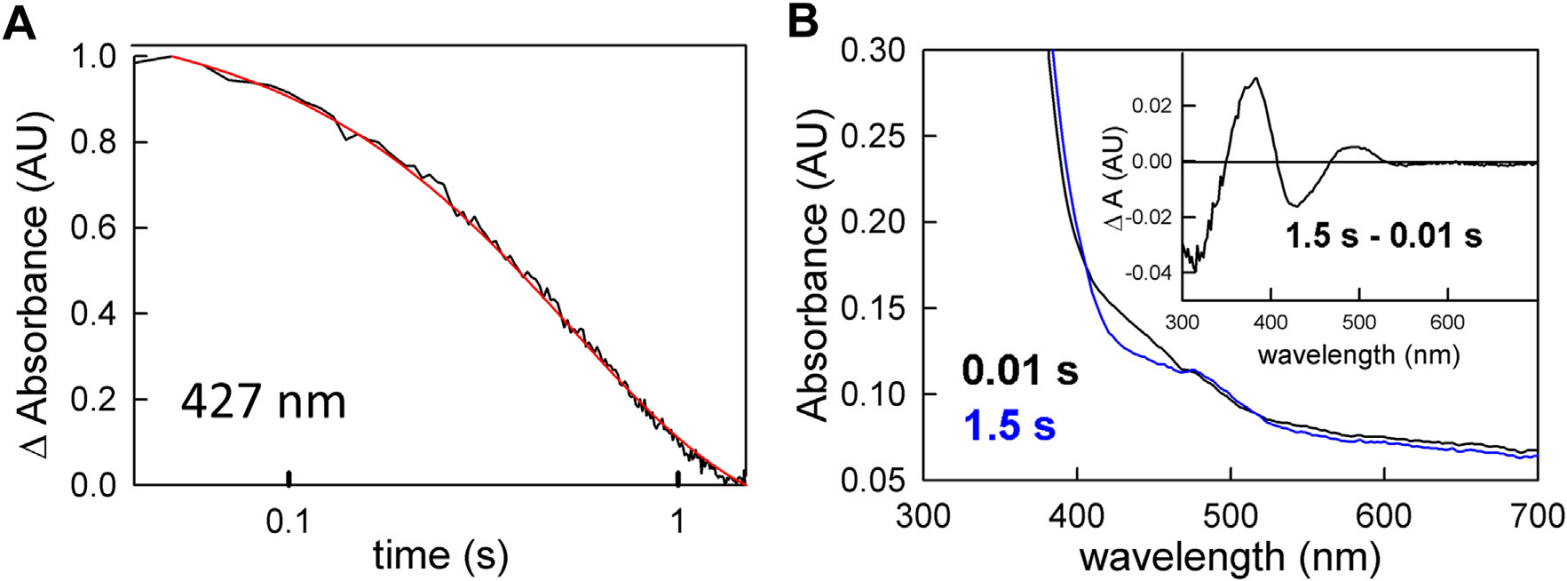
**Stoichiometric reduction of ferredoxin with post-catalytic EtfAB:bcd.***A*, the 427 nm transient (normalized to the total ΔA) indicative of ferredoxin reduction (*black*) and the overlayed exponential fit of 1.8 s^−1^ (*red*) for 5 μM EtfAB:bcd first reacted with 90 μM NADH and 50 μM crotonyl-CoA and subsequently reacted with 90 μM NADH, 50 μM crotonyl-CoA and 5 μM ferredoxin. *B*, rapid-reaction kinetics showing the spectral changes for the different phases of the reaction: the first observed spectrum at 0.01 s (*black*) and end of the reduction of ferredoxin 1.5 s (*blue*). Inset, the difference spectrum of 1.5 s minus 0.01 s (*black*) shows reduced ferredoxin and an appreciable amount of semiquinone formation as evident from the negative 420 nm and the positive 377 nm features, respectively. Experiments were performed in 50 mM Tris HCl, 150 mM NaCl, pH 7.5, 25 °C.

The above experiments were performed with a stoichiometric excess of ferredoxin to EtfAB:bcd (typically 3-fold) to ensure that binding of the oxidized ferredoxin to EtfAB:bcd was not an issue in the rate of reduction. Reacting the post-catalytic complex with a stoichiometric concentration of ferredoxin, however, allows for the monitoring of a single turnover. The greatest difference observed is that absent a pronounced lag, ferredoxin reduction (Fig. 10*A*) becomes essentially exponential, with a rate constant of 1.8 s^-1^ (Fig. 11*A*, red transient). This is comparable to the observed rate constant for electron transfer between the et FAD of EtfAB and the bcd FAD within the EtfAB:bcd complex, as reported previously (11). The resulting absorbance changes reflect ferredoxin reduction concomitant with formation of anionic semiquinone, as reflected in the negative feature at 420 nm and positive feature at 377 nm in the difference spectrum for the reaction, respectively (Fig. 11*B*, inset). This is confirmed in Fig. S3, where the subtraction between isolated semiquinone and ferredoxin yields a very similar difference spectrum to the Figure 11*B* inset. This demonstrates that accumulation of semiquinone is directly linked to ferredoxin reduction during catalysis. This directly linked semiquinone is likely from the et FAD, however, there are spectral contributions from a semiquinone on the bcd FAD as well, because the electron transfer that creates the et FAD•^-^ necessitates creation of the bcd FAD•^-^.

## Discussion

The intact EtfAB:bcd complex exhibits kinetic behavior and oxidation-reduction properties consistent with those of the isolated EtfAB and bcd components investigated in previous work (10, 11). For the reductive half-reaction, the initial reduction of the bf FAD by NADH is followed by a much slower further reduction that likely represents an intermolecular transfer of the low-potential electron in bf FAD•^-^ generated in the course of bifurcation to another molecule of the complex. Significantly, we find that the uncrossing of the bf FAD half-potentials observed with isolated EtfAB (10) is also observed in the intact EtfAB:bcd complex. This uncrossing makes full reduction of the complex difficult, with some FAD•^-^ and FADH• persisting even in the presence of excess NADH, and appears likely to be a safety valve mechanism whereby strongly reducing equivalents from bifurcation can be safely stored in the absence of ferredoxin, reducing the rate of intermolecular electron transfer (which would uncouple reduction of the low- and high-potential electron acceptors and defeat bifurcation).

For the oxidative half-reaction, the reaction of fully reduced EtfAB:bcd_6e_- with crotonyl-CoA is multiphasic, with a [crotonyl-CoA]-independent fast phase reflecting rapid reoxidation of the bcd FAD at a rate identical to that seen with the isolated bcd (11). This is followed by slower processes that involve transfer of a single electron from the et FADH^-^ to the now reoxidized bcd FAD, forming FAD•^-^ at both sites. Further reoxidation is incomplete, with a significant amount of FAD•^-^ persisting at the end of the reaction even in the presence of excess crotonyl-CoA; this is a consequence of the difficulty of fully reoxidizing the bf FAD in absence of ferredoxin. A portion of the incompletely reoxidized complex has a bf FAD with uncrossed half-potentials that is present as the anionic semiquinone. Thus, in contrast to the reaction with isolated bcd, reoxidation of the intact EtfAB:bcd complex is incomplete in the absence of the low-potential acceptor ferredoxin. In the presence of ferredoxin, on the other hand, reoxidation is more nearly complete (compare Figure 3, Figure 4*A*), as the presence of ferredoxin allows effective bifurcation and full reoxidation of the bf FAD in the course of the reaction. Still, the persistence of absorption in the 377 nm region at the end of the titration with crotonyl-CoA suggests that the et FAD remains partially reduced as the FAD•^-^ at the end of the titration, even in the presence of ferredoxin, consistent with it functioning between the SQ/HQ couple in the course of catalysis (see below).

The steady-state rate of reduction of ferredoxin by EtfAB:bcd has been reported to be 0.29 s^−1^ (12), much slower than either the initial reduction of the bf FAD by NADH or the reoxidation of the reduced bcd FAD by crotonyl-CoA. Using an enzyme-monitored turnover approach, we find two distinct phases in the course of the reaction of the enzyme with all three substrates under multiple turnover conditions. An initial approach to the steady-state phase occurs on a time scale similar to the 2 s^−1^ electron transfer from the bf FAD to the et FAD within EtfAB (10). There is no ferredoxin reduction in this phase of the reaction. This is followed by a steady-state phase in which EtfAB:bcd turns over all three substrates until the limiting substrate (ferredoxin) is consumed, with only very modest changes in the steady-state levels of flavin reduction in the complex but abundant reduction of ferredoxin (and presumably also oxidation of crotonyl-CoA to butyryl-CoA) at rates consistent with the previous published steady-state parameters (Fig. 7). Overall turnover, including bifurcation, appears to be rate-limited by the slow shuttling of electrons between the bf FAD and bcd FAD by the et FAD, which must occur four times for one complete turnover. As a result, the bf FAD is expected to be predominantly reduced throughout the steady-state. Consistent with rate-limiting electron transfer out of the bf FAD, varying the concentration of the high-potential acceptor crotonyl-CoA in these enzyme-monitored turnover experiments had little to no effect on the observed kinetics (Fig. 8).

In a single-turnover experiment with fully reduced EtfAB:bcd_6e_- and one equivalent of ferredoxin (but excess NADH and crotonyl-CoA), reduction of the low-potential acceptor occurs exponentially with a rate constant of 0.4 s^−1^ (Fig. 4). Although the initial phase seen in the enzyme-monitored turnover experiments persists in the observed kinetics, the spectral changes are significantly altered compared to the experiment with fully oxidized EtfAB:bcd and reflect a significant accumulation of FAD•^-^ in the course of the reaction. The duration of this initial phase is also significantly shortened with the reduced complex, indicating that there is some partially reduced state that the complex needs to achieve before ferredoxin reduction can begin. That this is the case is also demonstrated in enzyme-monitored turnover experiments with both EtfAB:bcd_1e_- (Fig. 9) and complex previously exposed to NADH and crotonyl-CoA (Fig. 10). In both cases the approach to steady-state phase of the reaction is significantly reduced or eliminated, with the onset of ferredoxin reduction occurring much earlier in the course of the reaction compared to the reaction with oxidized complex. That the photoreduced EtfAB:bcd_1e_- still exhibits an approach to steady-state phase with no ferredoxin reduction means that having a pre-reduced et FAD•^-^ is necessary but not sufficient for bifurcation as it is only with post-catalytic complex that the onset of ferredoxin reduction is immediate. The spectral change associated with this approach to steady-state phase is in fact mainly due to ferredoxin reduction, although features due to formation of anionic semiquinone are also present in the difference spectrum (Figs. 10*B* and 11*B*). It is clear that this post-catalytic form of the complex is “primed” for effective bifurcation and ferredoxin reduction, presumably by an unspecified conformational change (possibly associated with binding of ferredoxin at the bf FAD).

**Figure 12:**
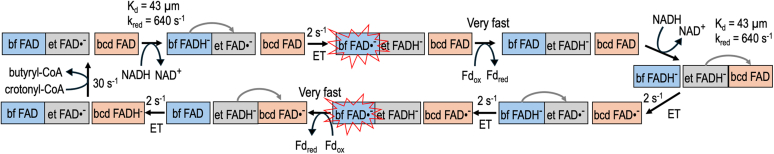
**A scheme for EtfAB:bcd in the course of turnover.** Starting with the “primed” form of the complex, with the et FAD (*gray*) in the semiquinone state, shown is the pathway that electrons take to be able to reduce ferredoxin. Electron transfer (ET) events between subunits are shown as *gray arrows*, and the red starburst demonstrates the instability of the bf FAD•^-^ due to the crossed potentials of the bf FAD.

The present results can be incorporated into a scheme delineating the oxidation states of the three flavins in the EtfAB:bcd system in the course of bifurcating turnover (Fig. 12). Present work is under way to determine the rate of the conformational change for the domain containing the et FAD and to assess the ability of ferredoxin to induce a bifurcation-ready conformation in EtfAB that enables bifurcation.

## Experimental procedures

### Organisms and growth conditions

#### EtfAB and bcd from *M. elsdenii*:

Two plasmids were constructed containing *M. elsdenii* EtfAB (*Mels_2126 + Mels_2127*) and bcd (*Mels_2128*) genes, both with a C-terminus Strep-II tag derived from pASG-IBA3 (IBA GmbH), as previously described by Chowdhury *et al.*, (12). These plasmids were transformed into *Escherichia coli* BL21 cells using growth conditions from Djordjevic *et al.*, (8). Cells obtained from glycerol stocks were put into 10 ml Terrific Broth (TB) alongside 100 μg/ml ampicillin and allowed to grow overnight at 37 °C with 220 rpm shaking. This preculture was then added to the 2 L of TB in a 6 L Erlenmeyer flask, again containing 100 μg/ml ampicillin for large-scale growth. This was grown for ∼6 h at 37 °C with 180 rpm shaking. As soon as the *A*_600 nm_ reached 0.5, the temperature was lowered to 21 °C. Shaking continued and the cells were induced with 0.1 mM anhydrotetracycline when *A*_600 nm_ reached 0.8 and left to grow overnight. Harvesting of cells was accomplished by centrifugation at 7500*g* at 4 °C for 20 min. The pellets were flash frozen using liquid nitrogen and then stored for later purification at −80 °C.

#### Ferredoxin from *M. elsdenii*:

A plasmid derived from a pTrcHisB vector with the 6xHis-linker-6xHis replaced with an N-terminal Strep-II tag was constructed containing the gene for *M*. *elsdenii* [4Fe-4S] ferredoxin (NCBI: locus tag C6Y28_RS04440). The plasmid was transformed into *E. coli* CLK002 cells (MG1655 *ΔiscR ΔmetJ*; Frank Sargent, Newcastle University, personal communication). Preculture consisted of cells obtained from glycerol stocks that were put into 80 ml TGYEP-rich media, with 0.8% glycerol and 100 μg/ml ampicillin. This was grown overnight at 30 °C with 220 rpm shaking. Enough preculture was then added to the large-scale growth, 3 L of TGYEP in a 6 L Erlenmeyer flask, again containing 100 μg/ml ampicillin, to bring the starting *A*_600 nm_ to 0.2 and then allowed to grow aerobically at 30 °C with 180 rpm shaking. As soon as the *A*_600 nm_ reached 1.5 to 1.8, cells were transferred to a 3 L flask for anaerobic growth. 2 mM IPTG and 100 μl of Antifoam B emulsion (Sigma-Aldrich), along with 0.25 g/L of L-cysteine and 0.2 g/L of ferric ammonium citrate were added to the cells after transfer. Flasks were sealed and constantly bubbled with UHP-grade argon as cells were allowed to grow anaerobically at room temperature with stirring. Harvesting of cells was accomplished by centrifugation at 7500*g* at 4 °C for 20 min. The pellets were flash frozen using liquid nitrogen and then stored for later purification at −80 °C.

#### Purification of *M. elsdenii* EtfAB, bcd, and Fd

Purification of EtfAB and bcd was performed aerobically between 0 to 4 °C. This procedure is a modified version of our previously described purification (11). The purification of both proteins was identical except for the buffers used, and the additional reduction of bcd to remove a persulfide CoA adduct. EtfAB used Buffer A (50 mM Tris-HCl, 150 mM NaCl, pH 7.5) and bcd used Strep-storage buffer (50 mM Tris, 150 mM NaCl, pH 8.0). The cells were resuspended in the corresponding buffer, with the addition of 1 mM NaF, 1 mM benzamidine, 0.5 mM phenylmethylsulfonyl fluoride (PMSF), and catalytic amounts of DNase I and lysozyme. The cells were incubated for 1 h before breaking. Lysis of the cells was achieved using 1 to 2 passes through a French Pressure Cell at 10,000 psi. Cell debris was removed from the lysate by centrifugation at 200,000*g* for 1 h. Supernatant was collected and flash frozen in liquid nitrogen and stored at −80 °C until further purification. Thawed supernatant was bound *via* batching with 6 ml of Strep-Tactin XT Superflow resin (IBA GmbH) pre-equilibrated with the corresponding buffer. The column was washed with 30 column volumes of buffer and 100 μM FAD, then eluted with 50 mM biotin in 50 mM Hepes (pH 7.5) at 0.5 ml/min. Elution was concentrated to a volume of less than 1 ml using an Amicon Ultra – 4 (EMD Millipore) centrifugal concentrator. To remove the protein-bound persulfide CoA (16), bcd was made anaerobic as described by Vigil *et al.* (13) with five vacuum cycles on an argon train. Sodium dithionite was added to completely reduce the bcd, then the protein was centrifuged as 10,000*g* for 30 min. To remove excess FAD and/or dithionite and buffer exchange both proteins into buffer A, an 8.3-mL PD-10 (Cytiva) equilibrated with buffer A was used. Purified protein was aliquoted, flash frozen, and stored at −80 °C until use.

Purification of Fd was performed semi-anaerobically. Cell lysis was performed at 4 to 10 °C in an anaerobic chamber (Coy Labs) with an atmosphere containing 2.5 to 3% H_2_. Frozen cells were thawed and resuspended in 50 mM Tris–HCl, 150 mM NaCl, pH 8.0 supplemented with 1 mM NaF, 1 mM benzamidine, 1 mM PMSF, 10 mM glucose, lysozyme, DNase I, and catalytic amounts of glucose oxidase and catalase. Cells were incubated for 1 h before breaking, then passed through an EmulsiFlex-B15 cell homogenizer (Avestin, Inc). Cell debris was removed by centrifugation at 200,000*g* for 30 min, and supernatant was flash frozen and stored in liquid nitrogen until purification. Column equilibration, binding, washing and eluting was performed outside of the glovebox at 0 to 4 °C with anaerobic buffers supplemented with 10 mM glucose and catalytic amounts of glucose oxidase from *Aspergillus niger* (Sigma Type VII, typically 80 nM) and bovine liver catalase (Sigma C-40, typically 8 nM) to scavenge residual O_2_ (10). A 5 ml Strep-Tactin XT Superflow column was equilibrated with 10 column volumes of 50 mM Tris–HCl, 150 mM NaCl, pH 8.0, and thawed supernatant was loaded at a rate of 0.5 ml/min onto the column. The column was washed with 20 column volumes of 50 mM Hepes, pH 7.5. Bound protein was eluted with 50 mM Hepes, 50 mM biotin pH 7.5 directly onto a 1 ml Q-Sepharose column (Cytiva). The Q-Sepharose column was washed with 20 column volumes of 50 mM Hepes pH 7.5, and then the bound protein was eluted with 50 mM Hepes, 0.5 M NaCl, pH 7.5. The purified ferredoxin was concentrated in Amicon Ultra - 4 concentrator and moved into anaerobic chamber to aliquot and then flash frozen and stored in liquid nitrogen until use.

#### UV-visible absorbance measurements

Static anaerobic titrations and general absorbance measurements were performed with a Hewlett-Packard 8452A diode-array spectrophotometer equipped with a thermostatted cell holder. Calculations of the concentrations of EtfAB and bcd were obtained using molar extinction coefficients of ε_450 nm_ = 21.1 mM^−1^ cm^−1^ and ε_450 nm_ = 11.8 mM^−1^ cm^−1^, respectively (7). Ferredoxin concentrations were obtained using ε_390 nm_ = 17.4 mM^−1^ cm^−1^ (3). Titrations were performed in a quartz anaerobic sidearm cuvette sealed with a rubber septum. Anaerobiosis of protein samples was achieved with an argon dry train on ice and contained glucose oxidase and catalase. After the sample was made anaerobic, glucose from the sidearm was added to the solution to a final concentration of ∼10 mM, to scrub the sample of any residual O_2_ as previously described (8). The reductant or oxidant, NADH (Thermo Scientific J61638.03), sodium dithionite (Reagents C2316300), or crotonyl-CoA (Sigma C6146) would also be made anaerobic on the argon train. The substrates were added to the sealed sidearm cuvette using a Hamilton syringe piercing the rubber septum. Apiezon N vacuum grease was used to seal the needle-septum interface. During a titration, an initial oxidized/reduced spectrum would be taken. After each addition of the substrate, a spectrum would be taken immediately, then another taken 5 to 10 min after addition to ensure a complete reaction. Substrate was added until the sample was fully oxidized/reduced or until no further reaction occurred.

#### Rapid-reaction kinetics

Rapid-reaction kinetics were performed using an Applied Photophysics SX-20 stopped-flow spectrophotometer equipped with either a photodiode array (PDA) or photomultiplier tube (PMT) detection with the acquisition software ProData SX 2.2.5.6. Since bcd is relatively unstable compared to the other proteins used, samples containing bcd were made anaerobic by first making buffer A and glucose anaerobic in a septum-sealed tonometer on an argon wet train, then a small volume of aerobic protein (including catalytic amounts of glucose oxidase and catalase) are injected by piercing the septa with a Hamilton syringe. This is then placed on ice for at least 30 min for the glucose oxidase and catalase to scavenge any residual O_2_ in the sample. Then, the sample is mounted onto the stopped-flow. Samples without bcd are made anaerobic on an argon wet train in a tonometer with catalytic amounts of glucose oxidase and catalase. After anaerobiosis is achieved, glucose is added from the sidearm to scavenge any residual O_2_ before mounting onto the stopped-flow. To generate EtfAB_1e-_, the sample was made anaerobic as described with the inclusion of a sidearm cuvette, and the protein was placed in front of a light source (lamp or sunlight) and the level of reduction was monitored spectrophotometrically until no further reaction was apparent at which point the tonometer was loaded onto the stopped-flow. To generate the fully reduced EtfAB:bcd_6e-_, protein was made anaerobic on an argon dry train on ice in a septum-sealed vial with catalytic amounts of glucose oxidase and catalase. This was then transferred into an anaerobic chamber and 10 mM glucose was added to scavenge residual O_2_. The sample was titrated to full reduction using dithionite with a StellarNet Inc Chem-System spectrophotometer and loaded into syringes; the syringes were then taken out of the anaerobic chamber and immediately mounted onto the stopped-flow. The photodiode array detector allows for the collection of absorbance in the 230 to 740 nm range. Extraction of a single wavelength from the dataset gives a kinetic transient used for rate determination. For NADH reduction, the photomultiplier tube was used due to the reaction being too fast for the photodiode array. Most experiments were conducted at 25 °C, except for the fast reactions, which were performed at 10 °C. The first 10 to 500 ms after mixing were used for analysis of the fast phase of FAD reduction. Time courses were fit to the sum of two exponentials by a nonlinear least squares regression analysis using the equation:

(1) $$A_{t}={\;A}_{\infty }\;\pm \sum A_{n}\;\exp\left(-t/k_{n}\right)$$

The number of kinetic phases is represented by n. Analysis of time courses was accomplished by ProData Viewer 4.2.0 software. The following equation was used to determine the limiting rate constant for reduction k_red_, and the apparent dissociation constant, K_d_ by plotting [substrate, S] against k_obs_, the observed rate constant:

(2) $$k_{\text{obs}}={\;k}_{\text{red}}\;\left[S\right]/\left(K_{d}+\left[S\right]\right)$$

The fast phase of EtfAB:bcd reduction (bf FAD) by NADH and the fast phase of EtfAB:bcd oxidation (bcd FAD) by crotonyl-CoA were fit to a single exponential. To determine the rate of the steady-state reduction of ferredoxin (k_cat_), the linear portion of the kinetic traces extracted from the PDA dataset at 427 nm was analyzed using a linear fit. To determine the different phases of ferredoxin reduction, both the kinetic transients and difference spectra were used. Since the reduction of ferredoxin was slow and required the protein to be exposed to the lamp for much longer, creating problems such as the photoreduction of the et FAD and the photodegradation of the bcd FAD. To avoid this, the stopped-flow was set up for a longer 10 ms integration time (opposed to the standard 1 ms time), allowing for the protein to be exposed to less light and for data collection to occur on longer timescales without modifying the proteins in any way.

## Data availability

Most data for this manuscript are present in the manuscript itself; raw and unprocessed data (absorption and EPR spectra, kinetic transients) are archived in the laboratory of the corresponding author and available on request.

## Supporting information

This article contains supporting information.

## Conflict of interest

The authors declare that they have no conflicts of interest with the contents of this article.

## Footnotes

† Both components of the present system had been individually purified and characterized before recognition that they were components of an electron-bifurcating system that functioned together. The EtfAB component was recognized as early as 1974 as containing two equivalents of FAD rather than just one (17, 18). Similarly, the crotonyl-CoA-reducing component was isolated and characterized in 1982 and termed butyryl-CoA dehydrogenase due to its functional (19) and structural (8) resemblance to mammalian medium-chain acyl-CoA dehydrogenases. It was only relatively recently that the two components functioned together as a crotonylCoA-dependent NADH:ferredoxin oxidoreductase (12). The historical designation of the crotonylCoA-reducing component as butyryl-CoA dehydrogenase is retained here although it functions physiologically in the opposite direction.

## References

[1] Buckel W., Thauer R.K. (2018). Flavin-based electron bifurcation, A new mechanism of biological energy coupling. Chem. Rev..

[2] Buckel W., Thauer R.K. (2018). Flavin-based electron bifurcation, ferredoxin, flavodoxin, and anaerobic respiration with protons (Ech) or NAD(+) (Rnf) as electron acceptors: a historical review. Front. Microbiol..

[3] Herrmann G., Jayamani E., Mai G., Buckel W. (2008). Energy conservation via electron-transferring flavoprotein in anaerobic bacteria. J. Bacteriol..

[4] Ortiz S., Niks D., Wiley S., Lubner C.E., Hille R. (2023). Rapid-reaction kinetics of the bifurcating NAD plus -dependent NADPH:ferredoxin oxidoreductase NfnI from *Pyrococcus* furiosus. J. Biol. Chem..

[5] Demmer J.K., Chowdhury N.P., Selmer T., Ermler U., Buckel W. (2017). The semiquinone swing in the bifurcating electron transferring flavoprotein/butyryl-CoA dehydrogenase complex from Clostridium difficile. Nat. Commun..

[6] Toogood H.S., van Thiel A., Basran J., Sutcliffe M.J., Scrutton N.S., Leys D. (2004). Extensive domain motion and electron transfer in the human electron transferring flavoprotein•medium chain acyl-CoA dehydrogenase complex. J. Biol. Chem..

[7] Toogood H.S., Leys D., Scrutton N.S. (2007). Dynamics driving function - new insights from electron transferring flavoproteins and partner complexes. Febs J..

[8] Djordjevic S., Pace C.P., Stankovich M.T., Kim J.J.P. (1995). 3-Dimensional structure of butyryl-CoA dehydrogenase from *Megasphaera elsdenii*. Biochemistry.

[9] Kim J.J.P., Wu J. (1988). Structure of teh medium-hcain acylCoA dehydrogenase from pig liver mitochondria at 3 Å resolution. Proc. Natl. Acad. Sci. USA.

[10] Vigil W., Tran J., Niks D., Schut G.J., Ge X.X., Adams M.W.W. (2022). The reductive half-reaction of two bifurcating electron-transferring flavoproteins: evidence for changes in flavin reduction potentials mediated by specific conformational changes. J. Biol. Chem..

[11] Vigil W., Nguyen D., Hille R. (2023). Rapid Reaction Kinetics & characterization of FAD in the reductive and oxidative half reactions of bcd in Megasphaera elsdenii. J. Biol. Chem..

[12] Chowdhury N.P., Kahnt J., Buckel W. (2015). Reduction of ferredoxin or oxygen by flavin-based electron bifurcation in Megasphaeraelsdenii. FEBS J..

[13] Vigil W., Nguyen D., Niks D., Hille R. (2023). Rapid-reaction kinetics of the butyryl-CoA dehydrogenase component of the electron-bifurcating crotonyl-CoA-dependent NADH:ferredoxin oxidoreductase from Megasphaera elsdenii. J. Biol. Chem..

[14] Hille R. (1996). The mononuclear molybdenum enzymes. Chem. Rev..

[15] Penzer G.R., Radda G.K. (1968). Chemistry of flavines and flavoproteins - photoreduction of flavines by amino aciids. Biochem. J..

[16] Williamson G., Engel P.C., Mizzer J.P., Thorpe C., Massey V. (1982). Evidence that the greening ligand in native butyryl-CoA dehydrogenase is a CoA persulfide. J. Biol. Chem..

[17] Mayhew S.G., Whitfield C.D., Ghisla S., Schumanj M. (1974). Identification and properties of new flavins in electron-transferring flavoprotein from *Peptostreptococcus elsdenii* and pig liver glycolate oxidase. Eur. J. Biochem..

[18] Whitfield C.D., Mayhew S.G. (1974). Purification and properties of electron-transferring flavoprotein from *Peptostreptococcus elsdenii*. J. Biol. Chem..

[19] Williamson G., Engel P.C. (1984). Butyryl-Co dehydrognase from *Megasphaera elsdenii/*. Specificity of the catalytic reaction. Biochem. J..

