# Supplementary material for: The rapid-reaction kinetics of an electron-bifurcating flavoprotein, the crotonyl-CoA-dependent NADH:ferredoxin oxidoreductase EtfAB:bcd
Source: J Biol Chem. 2024 Sep 3;300(10):107745. doi: 10.1016/j.jbc.2024.107745 (PMC11480532; doi:10.1016/j.jbc.2024.107745)
Supplement: Supporting Information [file mmc1.docx]

**
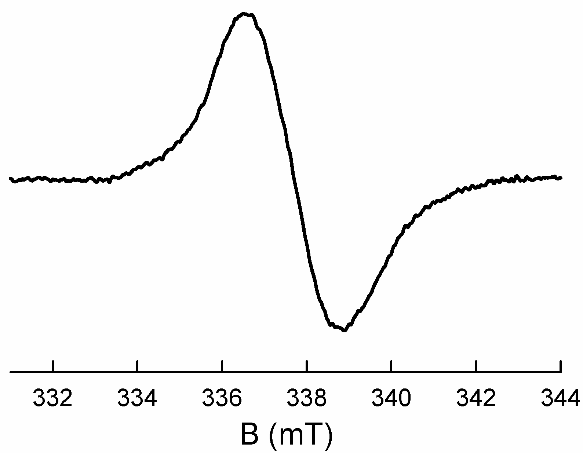
**

**Figure S1.** *77 K EPR of EtfAB:bcd reduction by NADH at short timescales in the presence of crotonyl-CoA*. Spectrum of 16 µM EtfAB:bcd, reacted with an excess (250 µM) of NADH and 100 µM crotonyl-CoA and frozen at ~0.5 s after mixing. The rapid formation of anionic semiquinone (1.6 mT) must be due to the uncrossing of the bf FAD, producing semiquinones on both the bf and et FADs, which has been reported in the isolated EtfAB NADH reduction. Rapid freezing was achieved by chilling an anaerobic solution of EtfAB:bcd in a sealed EPR tube on ice, then after injecting and mixing the solution of NADH and crotonyl-CoA (via Hamilton syringe), the tube was quickly frozen in a dry ice/ethanol bath. EPR spectra were observed using a Bruker Magnettech ESR5000 spectrometer with 0.02 mW microwave power and 0.6 mT modulation amplitude.

**

**

**Figure S2.** *Reduction of EtfAB by light and NADH*. Spectra of 12 µM EtfAB, exposed to light and then reacted with 290 µM NADH. Spectra shown at different times after exposure to light and immediately after mixing with excess NADH are as follows: oxidized (black), 1 minute (blue), 3 minutes (green), 5 minutes (red) and ~ 1 s after addition of 290 µM NADH (black). The lack of significant changes in absorption after 1 minute demonstrated that the extent of photoreduction was complete by 5 minutes and that FAD•- did not transfer electrons to the other FAD, indicating only one FAD was photoreduced. After reaction with excess NADH, immediate reduction of the remaining FAD is proof that the oxidized FAD after photoreduction was unequivocally the bf FAD and that the et FAD•- was the species observed. Inset, difference spectra of 5 minutes of light exposure minus oxidized (black) and NADH reduced minus 5 minutes of light exposure (red) demonstrating the concomitant reduction of the et FAD and formation of et FAD•- as well as the reduction of bf FAD to FADH-. Photoreduction of EtfAB was performed in 50 mM Tris HCl, 150 mM NaCl, pH 7.5 in anaerobic glass cuvettes. After anaerobiosis of samples was achieved, samples were either exposed to direct sunlight on ice at 1-minute intervals.


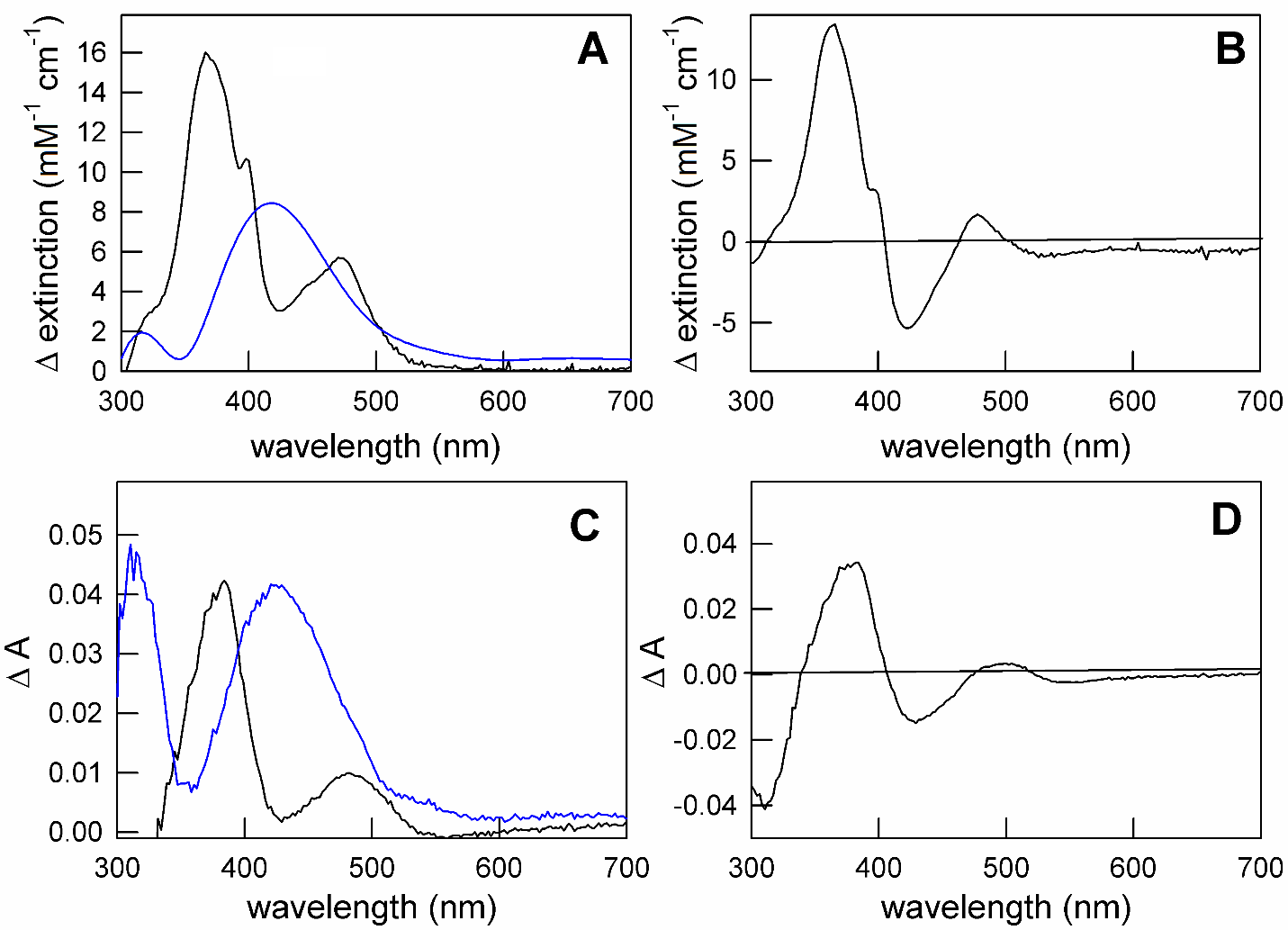


**Figure S3.** *Isolated difference spectra of FAD*•- and *ferredoxin*. Panel A, difference spectra showing a nearly pure spectrum of FAD•- (black) and oxidized minus reduced ferredoxin (blue), both constructed from anaerobic titrations with dithionite of *M. elsdenii* of EtfAB and ferredoxin, respectively. Panel B, double difference spectrum of FAD•- minus ferredoxin (from Panel A) showing the contribution of the 377 nm maximum of the anionic semiquinone (positive feature) and the clear negative minimum of the reduced ferredoxin centered ~ 410 nm. Panel C, the extracted difference spectra of isolated 2.5 µM FAD•- (black) and 5 µM reduced ferredoxin (blue) from experiments performed in the stopped-flow apparatus depicted in Figure 11. Panel D, double difference spectrum of FAD•- minus ferredoxin from Panel C in a 1:2 ratio to mimic experimental conditions in Figure 11, showing again the 377 nm maxima of oxidized FAD•- and the 410 nm minima of reduced ferredoxin.
